# Regulation of 5-oxo-ETE synthesis by nitric oxide in human polymorphonuclear leucocytes upon their interaction with zymosan and *Salmonella typhimurium*

**DOI:** 10.1042/BSR20130136

**Published:** 2014-05-23

**Authors:** Galina M. Viryasova, Svetlana I. Galkina, Tatjana V. Gaponova, Julia M. Romanova, Galina F. Sud’ina

**Affiliations:** *Lomonosov Moscow State University, Belozersky Institute of Physico-Chemical Biology, Moscow 119234, Russia; †FGBU Hematology Research Centre, Russia Federation Ministry of Public Health, Moscow 125167, Russia; ‡Gamaleya Research Institute of Epidemiology and Microbiology, Moscow 123098, Russia

**Keywords:** leukotriene, lipoxygenase, neutrophil, nitric oxide, 5-oxo-ETE, *Salmonella*, 5-LOX, 5-lipoxygenase, 5-HEDH, 5-hydroxyeicosanoid dehydrogenase, 5-HETE, 5S-hydroxy-6,8,11,14-eicosatetraenoic acid, 5-oxo-ETE, 5-oxo-6,8,11,14-eicosatetraenoic acid, AA, arachidonic acid, ANF, atrial natriuretic peptide, DEA NONOate, 1,1-diethyl-2-hydroxy-2-nitroso-hydrazine sodium, Dnp-Cl, 1-chloro-2,4-dinitrobenzene, G-CSF, granulocyte colony-stimulating factor, GM-CSF, granulocyte/macrophage colony-stimulating factor, HBSS, Hanks balanced salt solution, HBSS/Hepes, Hanks balanced salts medium containing 10 mM Hepes, G6PD, glucose-6-phosphate dehydrogenase, LPS, lipopolysaccharide, LT, leukotriene, LTB_4_, leukotriene B_4_, mGC, membrane-bound form of GC, NO, nitric oxide, PGB_2_, prostaglandin B_2_, PMA, phorbol 12-myristate 13-acetate, PMNL, polymorphonuclear leucocyte, OZ, opsonized zymosan, ROS, reactive oxygen species, sGC, soluble guanylyl cyclase, YC-1, 3-(5′-hydroxymethyl-2′-furyl)-1-benzylindazole

## Abstract

In the present study we have presented data on the regulation of LT (leukotriene) and 5-oxo-ETE (5-oxo-6,8,11,14-eicosatetraenoic acid) syntheses in human neutrophils upon interaction with OZ (opsonized zymosan) or *Salmonella typhimurium*. Priming of neutrophils with PMA (phorbol 12-myristate 13-acetate) and LPS (lipopolysaccharide) elicits 5-oxo-ETE formation in neutrophils exposed to OZ, and the addition of AA (arachidonic acid) significantly increases 5-oxo-ETE synthesis. We found that NO (nitric oxide)-releasing compounds induce 5-oxo-ETE synthesis in neutrophils treated with OZ or *S. typhimurium.* Exposure of neutrophils to zymosan or bacteria in the presence of the NO donor DEA NONOate (1,1-diethyl-2-hydroxy-2-nitroso-hydrazine sodium) considerably increased the conversion of endogenously formed 5-HETE (5S-hydroxy-6,8,11,14-eicosatetraenoic acid) to 5-oxo-ETE. To our knowledge, this study is the first to demonstrate that NO is a potent regulator of 5-oxo-ETE synthesis in human polymorphonuclear leucocytes exposed to *Salmonella typhimurium* and zymosan.

## Abstract 2

Nitric oxide significantly increased 5-oxo-ETE formation in neutrophils. 5-oxo-ETE is a key 5-lipoxygenase metabolite in human polymorphonuclear leucocytes exposed to NO upon interaction with opsonized zymosan or *Salmonella typhimurium.*

## INTRODUCTION

**Scheme 1:**
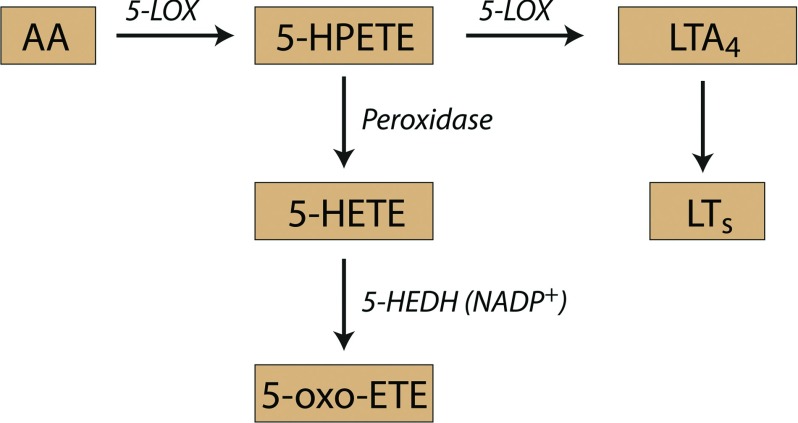
Schematic presentation of leukotriene and 5-oxo-ETE synthesis.

The 5-LOX (5-lipoxygenase) pathway converts AA (arachidonic acid) to 5-HETE (5S-hydroxy-6,8,11,14-eicosatetraenoic acid) and LTs (leukotrienes). The 5-LOX pathway in neutrophils metabolizes AA to LTB_4_ (leukotriene B_4_), which mediates host defence and inflammatory responses [1]. 5-LOX is activated during the phagocytosis of microorganisms and foreign particles by neutrophils [2], which is accompanied by a considerable increase in NADPH-oxidase activity that generates superoxide anion O_2_^−^ [3]. Simultaneously, neutrophils produce an arsenal of regulatory molecules such as chemokines and cytokines [4]. One branch of the 5-LOX pathway produces 5-oxo-ETE (5-oxo-6,8,11,14-eicosatetraenoic acid) [5], which is the most potent eosinophil chemoattractant among lipid mediators [6,7] and exerts the similar effects on neutrophils [8,9] and monocytes [10]. 5-oxo-ETE is a potent stimulator of calcium mobilization [8] and surface expression of integrins in neutrophils [11]. 5-oxo-ETE inhibits apoptosis in prostate cancer cell lines [12,13] and in neuroblastoma cell lines [14]. 5-oxo-ETE is formed by the oxidation of the 5-LOX product 5-HETE by 5-HEDH (5-hydroxyeicosanoid dehydrogenase) (Scheme 1). 5- HEDH is highly selective for this substrate and requires NADP^+^ as a cofactor [15]. Despite the high level of 5-HEDH activity in the microsomes of neutrophils, intact cells convert 5-HETE to only trace amounts of 5-oxo-ETE, suggesting that the production of this substance is highly regulated [5].

Activation of the respiratory burst by PMA (phorbol 12-myristate 13-acetate) in phagocytic cells increases the rate of formation of 5-oxo-ETE [16]. Non-enzymatic conversion of intracellular NADPH to NADP^+^ by the addition of phenazine methosulfate activates 5-oxo-ETE synthesis from exogenously added 5-HETE, suggesting that the stimulatory effect of NADPH oxidase activation is due to the generation of NADP^+^, the cofactor required by 5-HEDH [17]. Most of the data on 5-oxo-ETE synthesis have been obtained using PMNLs (polymorphonuclear leucocytes) stimulated with the calcium ionophore A23187 or from exogenously added 5-HETE. Serum-treated zymosan stimulates the conversion of exogenously added 5-HETE to 5-oxo-ETE in neutrophils [17]; however, to our knowledge, no data are available on the production of 5-oxo-ETE by neutrophils when they are engaged in phagocytizing a target.

Therefore we focused our research on the regulation of 5-oxo-ETE synthesis in PMNLs exposed to OZ (opsonized zymosan) or *Salmonella typhimurium*. We found that priming PMNLs with PMA or LPS (lipopolysaccharides), which activate ROS (reactive oxygen species) formation, induced 5-oxo-ETE synthesis upon interaction with *S. typhimurium* and zymosan. When 5-HETE synthesis was increased by adding AA, we observed a concomitant increase in 5-oxo-ETE formation. Only stimulation of PMNLs with zymosan or bacteria in the presence of an NO donor significantly increased the conversion of endogenously formed 5-HETE to 5-oxo-ETE.

## MATERIAL AND METHODS

The virulent strain of *S. typhimurium* (C53) was a kind gift from Professor F. Norel (Pasteur Institute, France) [18]. Bacteria [stock, 1×10^9^ CFU (colony-forming units)/ml] were grown in Luria–Bertani broth, washed twice by using a physiological salt solution, and collected by centrifugation at 2000 ***g***. The bacteria were opsonized using fresh serum from the same donor whose blood was used to prepare neutrophils. Serum was isolated by centrifuging the clotted blood. For opsonization, bacteria were incubated for 15 min in Dulbecco's salt solution containing 5% (v/v) serum. Bacteria were washed by repeated centrifugation in Dulbecco's solution. HBSS (Hanks balanced salt solution) with calcium and magnesium but without phenol red and sodium hydrogen carbonate, Dulbecco's PBS with magnesium but without calcium, Dnp-Cl (1-chloro-2,4-dinitrobenzene), diamide and LPSs from *Salmonella enterica* serovar *Typhimurium* (smooth form derived from the strain ATCC 7823, Ra mutant TV119 and Re mutant SL1181) were from Sigma-Aldrich. LPS from deep core mutant (Re), complete core (Ra) and smooth (S) phenotypes of *S. typhimurium* was used, as they differ in the ability to activate superoxide production in neutrophils [19]. YC-1 [3-(5′-hydroxymethyl-2′-furyl)-1-benzylindazole] and fibronectin were from Calbiochem. The NO (nitric oxide) donor DEA (diethylamine) NONOate (1,1-diethyl-2-hydroxy-2-nitroso-hydrazine sodium), 5-HETE, 5-HPETE and AA were from Cayman Chemical. Ficoll-Paque was purchased from Pharmacia. Zymosan A particles from *Saccharomyces cerevisiae* (Sigma) were boiled in PBS for 5 min and were opsonized by incubation with 30% (v/v) autologous human serum for 30 min at 37°C; they were then washed and resuspended in HBSS/Hepes (Hanks balanced salts medium containing 10 mM Hepes).

### Ethics statement

We prepared neutrophils from the blood of healthy volunteers. Blood was collected via venous puncture as approved by the Ministry of Public Health Service of the Russian Federation. Experimental and subject consent procedures were approved by the Institutional Ethics Committee of the A. N. Belozersky Institute of Physico-Chemical Biology.

### Isolation of PMNLs

PMNLs were isolated from freshly drawn citrated donor blood using the standard techniques [20]. Leucocyte-rich plasma was prepared by sedimenting erythrocytes through 3% (w/v) dextran T-500 at room temperature (22–24°C). Granulocytes were purified by centrifugation through Ficoll-Paque (density, 1.077 g/ml) followed by hypotonic lysis of the remaining erythrocytes. PMNLs were washed twice with PBS, resuspended at 10^7^ cells/ml (purity, 96–97%; viability 98–99%) in Dulbecco's PBS containing 1 mg/ml glucose (without CaCl_2_), and stored at room temperature.

### Incubations for studies of AA metabolism

PMNLs (2×10^7^ cells) were incubated in 6 ml HBSS/Hepes medium at 37°C with or without test compounds for 30 min and were treated with zymosan or bacteria for 15–60 min (see figure legends). The treatment was terminated by the addition of an equal volume of methanol at −20°C. PGB_2_ (prostaglandin B_2_) served as an internal standard. The samples were stored at −20°C. The denatured cell suspension was centrifuged, and the supernatants obtained were considered water/methanol extracts.

### Analysis of lipoxygenase reaction products

The water/methanol extracts were purified by solid-phase extraction by using C_18_ Sep-Paks (500 mg) that were first equilibrated with methanol and then with water. The metabolites of 5-LOX were extracted using 1.5 ml methanol, and the samples were evaporated and redissolved in 50 μl methanol/water (2:1). The purified samples were injected into a 5 μm, 250×4.6 mm Nucleosil® C18 column (Macherey-Nagel GmbH) and subjected to RP (reverse-phase) HPLC. The products were eluted at flow rate of 0.7 ml/min with a 30-min linear gradient from 20 to 70% solvent B, followed by a 3-min isocratic elution, and then elution for 3 min by using a linear gradient of 70–100% solvent B. The eluents consisted of methanol/acetonitrile/water/acetic acid/triethylamine in the ratios (A) 25:25:50:0.05:0.075 and (B) 50:50:0:0.05:0.04. Products of the 5-LOX pathway included 5S,12R-dihydroxy-6,14-*cis*-8,10-*trans*-eicosatetraenoic acid (LTB_4_), iso-LTB_4_ (5S,12SR-all-*trans*-diHETE), ω-OH- LTB_4_, 5S-hydroxy-6-*trans*-8,11,14-*cis*-eicosatetraenoic acid (5-HETE) and 5-oxo-ETE (5-oxo-6-*trans*-8,11,14-*cis*-eicosatetraenoic acid). They were identified by their co-elution with the standards. The compounds were quantified by comparing their peak areas with that of PGB_2_ and were corrected for differences in extinction coefficients as follows: PGB_2_, 27000; LTB_4_, iso-LTB_4_ and ω-OH-LTB_4_, 40 000 at 280 nm; 5-oxo-ETE, 22 000 at 279 nm and 5-HETE, 27 000 at 236 nm.

### Scanning electron microscopy (SEM)

Neutrophils were deposited on fibronectin-coated cover-slips for 20 min (control) or in the presence of PMA, DEA NONOate and OZ particles, as indicated in the figure legend. The cells were fixed for 30 min in 2.5% (w/v) glutaraldehyde, fixed again for 15 min with 1% (w/v) osmium tetroxide in 0.1 M cacodylate (pH 7.3), dehydrated using a series of acetone concentrations, subjected to critical point drying with liquid CO_2_ as the transitional fluid in a Balzers apparatus, sputter-coated with gold–palladium and observed at 15 kV by using a Camscan S-2 (Tescan, Cranberry Twp) SEM.

### Statistical analysis

Results have been reported as mean±S.D. Analysis of the statistical significance was performed by Student's *t* test using GraphPadPrism6 and SigmaPlot9.0 software. Differences with *P* values of <0.05 were considered statistically significant.

## RESULTS

### Priming of PMNLs with PMA and LPS elicits 5-oxo-ETE formation upon treatment with zymosan

**Figure 1:**
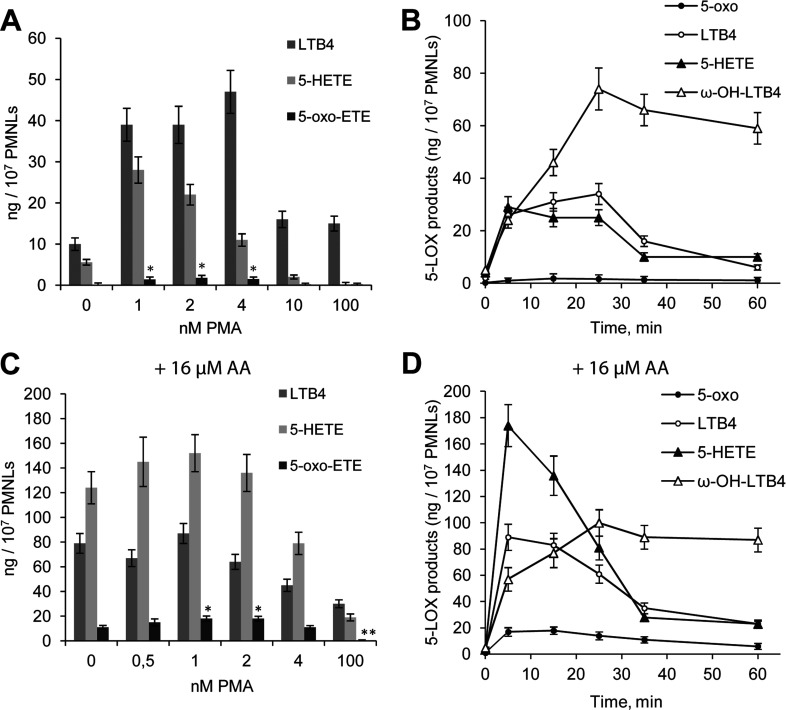
Effects of PMA concentration on LTB_4_, 5-HETE and 5-oxo-ETE production by PMNLs exposed to OZ (A) or AA+OZ (C) and time course of the 5-LOX reaction in the presence of 2 nM PMA (**B**,**D**) PMNLs (2×10^7^) were incubated for 30 min at 37°C without additives or with the indicated concentration of PMA and then 2 mg/ml OZ was added to the cells for 15 min (**A**,**C**) or for the indicated time (**B**,**D**). AA (16 μM) was added simultaneously with OZ (**C**,**D**). The products of the reaction catalysed by 5-LOX were extracted from the medium and separated using HPLC. The synthesis of all products of the 5-LOX pathway was analysed, and the data for LTB_4_, 5-HETE, 5-oxo-ETE and ω-OH-LTB_4_ are presented. Results (**A**,**C**) are means±S.D. of four independent experiments,*P<0.05, **P<0.01 versus the corresponding control without PMA. Results of time studies (**B**,**D**) are means±S.D. of two independent experiments performed in duplicate.

Human neutrophils synthesize 5-LOX metabolites (LTB_4_ and its isomers, ω-OH-LTB_4_ and 5-HETE) during the phagocytosis of OZ or *S. typhimurium* (OS) [19,21]. We incubated PMNLs in HBSS/Hepes. In the absence of other additives, PMNLs exposed to zymosan synthesized LTB_4_, its isomers, ω-OH-LTB4 and modest amounts of 5-HETE (Figure 1A). Preincubation (priming) of cells with PMA augmented the synthesis of 5-LOX products as well as the formation of 5-oxo-ETE during the interaction of PMNL with OZ. The maximum effect was observed in the presence of 2 nM PMA (Figure 1A). The accumulation of 5-oxo-ETE by PMNLs in response to zymosan lasted for 15 min (Figure 1B).

To exclude the possibility that the yields of 5-LOX products were limited by the availability of AA, we added 16 μM (optimum concentration, results not shown) AA together with OZ. There was a significant increase in 5-HETE and 5-oxo-ETE synthesis by PMNLs with maximal 5-oxo-ETE production at 1–2 nM PMA (Figure 1C). Neither PMA nor AA induced LT and 5-oxo-ETE syntheses in PMNLs in the absence of zymosan (results not shown). The accumulation of 5-oxo-ETE ceased 15 min after exposure of PMNLs to OZ and AA (Figure 1D). AA increases the oxidation state of the cell [22], and the increase in 5-oxo-ETE may be related to this effect. Furthermore, NO is a potent regulator of the oxidative state of cells. A large amount of NO is generated during inflammation (review in [23]).

### NO activates 5-oxo-ETE synthesis by human neutrophils upon their interaction with zymosan or *S. typhimurium*

**Figure 2:**
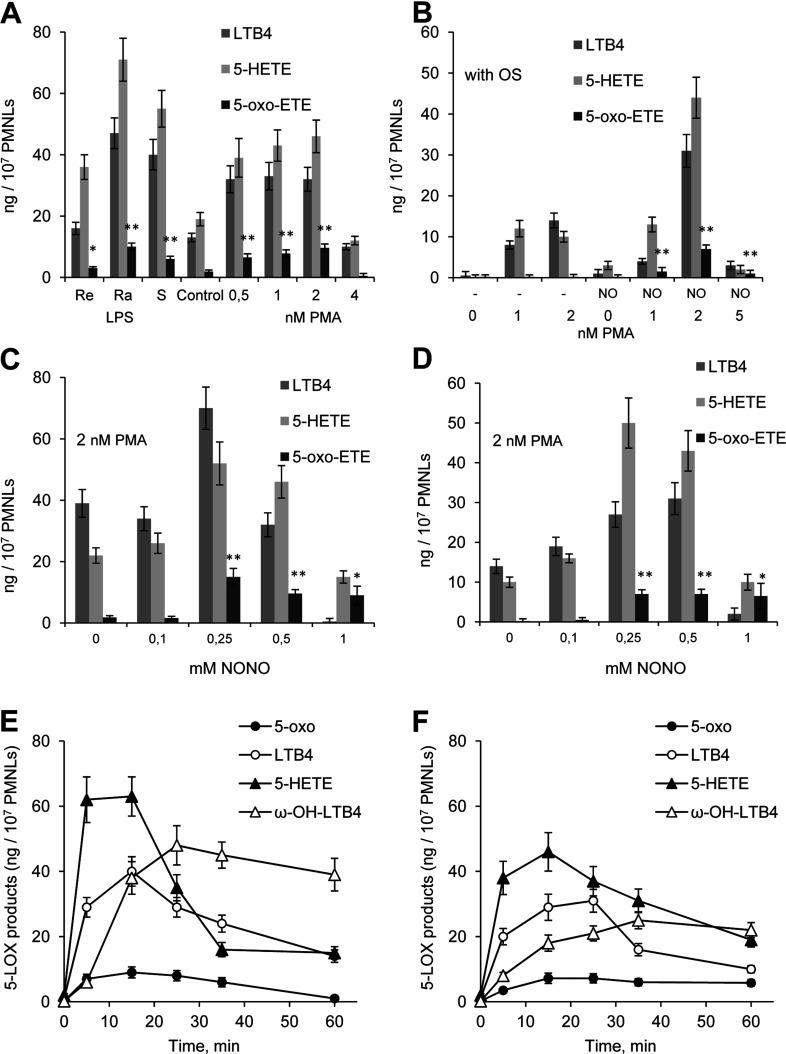
Effects of NO on the formation of 5-LOX reaction products and 5-oxo-ETE in human PMNLs (**A**,**B**) PMNLs (2×10^7^) were incubated for 30 min at 37°C without additives or with 1 μM of different LPS forms or PMA (1–5 nM, as indicated), and then 2 mg/ml of zymosan was added for 15 min (**A**) or 5×10^8^ *S. typhimurium* was added for 20 min (**B**). OZ or OS was added simultaneously with DEA NONOate (final concentration, 500 μM). The results represent the means±S.D. of four independent experiments, *P<0.05 and **P<0.01, compared with the corresponding controls without PMA or LPS. (**C**,**D**) PMNLs (2×10^7^) were incubated for 30 min at 37°C with 2 nM PMA, and then either 2 mg/ml OZ was added for 15 min (**C**) or 5×10^8^ OS was added for 20 min (**D**). OZ or OS was added simultaneously with DEA NONOate at the indicated final concentration of NONOate (0.1–1 mM). The results represent the means±S.D. of four independent experiments, *P<0.05 and **P<0.01, compared with the corresponding data without NO donor. (**E**,**F**) Time course of the 5-LOX reaction in PMNLs. PMNLs were incubated with 2 nM PMA for 30 min and then exposed to NO_OZ (**E**) or NO_OS (**F**) in a final concentration of 500 μM NONOate. The products generated by 5-LOX were extracted from the medium and separated using HPLC. The results are means±S.D. of three independent experiments performed in duplicate.

To determine whether NO directly affected 5-oxo-ETE synthesis in human neutrophils, we tested whether the NO-donor DEA NONOate influenced 5-oxo-ETE formation. Addition of the NO-donor DEA NONOate simultaneously with OZ (NO_OZ) induced 5-oxo-ETE synthesis in PMNLs that were not primed (Figure 2A, control). Priming of neutrophils with PMA and LPS (LPS at concentrations when the effect is maximal) efficiently increased the 5-oxo-ETE release, which was particularly apparent when the neutrophils interacted with opsonized *S. typhimurium* (Figure 2B). Among various LPS forms Ra-LPS possessing the maximal ability to activate superoxide production demonstrated the most prominent effect on 5-oxo-ETE synthesis (Figure 2A). Adding DEA NONOate to the bacteria caused a significant increase in the synthesis of 5-LOX products and 5-oxo-ETE during the interactions of neutrophils with the bacteria. We added various amounts of DEA NONOate with OZ or OS and found that NO_OZ was maximally effective in the presence of 250 μM NONOate (Figure 2C). The effects of 250 and 500 μM NONOate were comparable in the action of NO_OS (Figure 2D). Accumulation of 5-oxo-ETE ceased 15 or 20 min after the PMNLs were treated with NO_OZ (Figure 2E) or NO_OS (Figure 2F), respectively.

**Figure 3:**
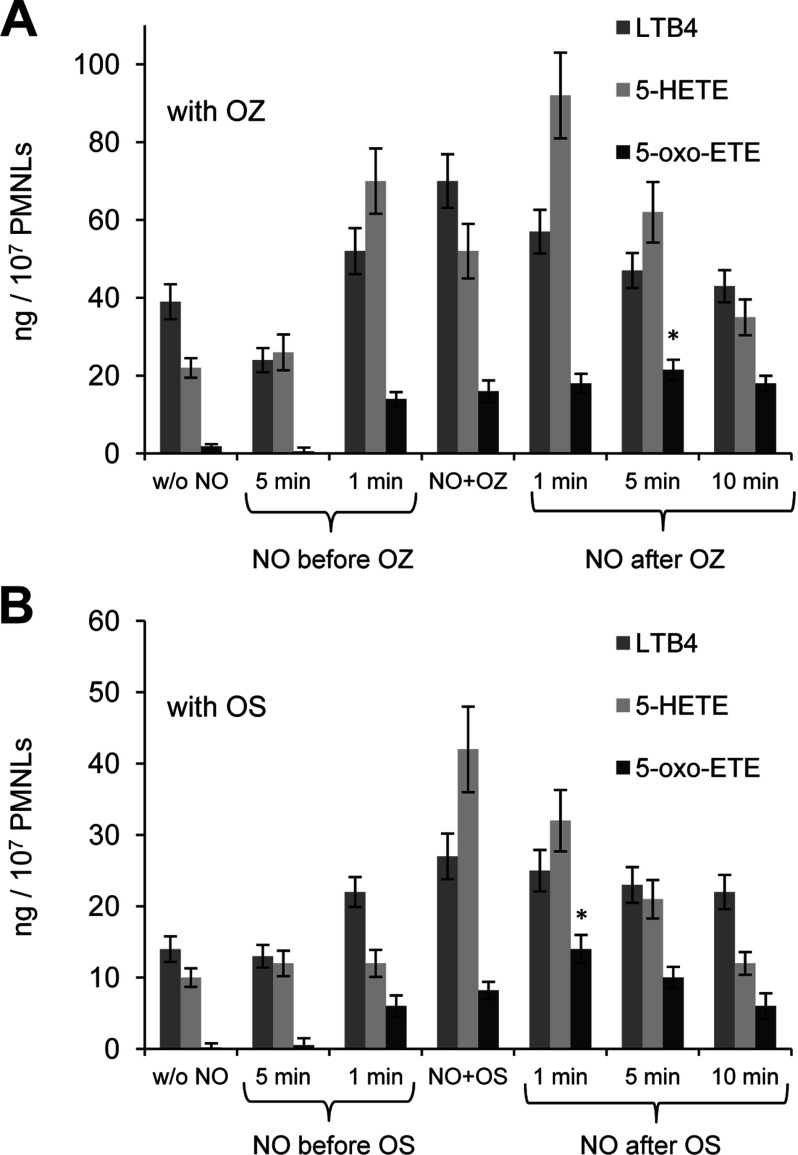
Effect of the NO donor DEA NONOate on LTB_4_, 5-HETE and 5-oxo-ETE syntheses in PMNLs (**A**) PMNLs (2×10^7^) were incubated for 30 min at 37°C with 2 nM PMA, and then for 15 min with 2 mg/ml OZ; 250 μM DEA NONOate was added before (5 and 1 min before), simultaneously (NO+OZ) or after OZ (1, 5 and 10 min after) as indicated. The results represent the means±S.D. of five independent experiments, *P<0.05, compared with data when the NO donor was added simultaneously with OZ. (**B**) PMNLs (2×10^7^) were incubated for 30 min at 37°C with 2 nM PMA and then for 15 min with 5×10^8^ OS. DEA NONOate (250 μM) was added before (5 and 1 min before), simultaneously (NO+OS), and after OS (1, 5, and 10 min after) as indicated. The results represent the means±S.D. of four independent experiments, *P<0.05, compared with data acquired when the NO donor added simultaneously with OS.

We next added DEA NONOate to PMNLs for different fixed times before their exposure to OZ or OS as well as after adding a target of phagocytosis (Figure 3). Formation of 5-oxo-ETE was maximized when the NO donor was added 5 min after the addition of OZ (Figure 3A) or 1 min after the addition of OS (Figure 3B). To achieve optimal synthesis of 5-oxo-ETE, it was important to add the NO donor after the initial interaction of PMNLs with OZ or OS. 5-HETE or 5-HPETE (5S-hydroperoxy-eicosatetraenoic acid) added directly with DEA NONOate did not give 5-oxo-ETE (results not shown). Adding to neutrophils of diamide or Dnp-Cl to increase the intracellular concen-tration of 5-HPETE [24,25] inhibited rather than increased the synthesis of 5-oxo-ETE (results not shown). We assume that a mechanism of 5-oxo-ETE formation by a free radical reaction of 5-HpETE with NO is hardly possible, so we propose enzymatic, 5-HEDH-catalysed conversion of 5-HETE to 5-oxo-ETE in the presence of NO donor.

**Figure 4:**
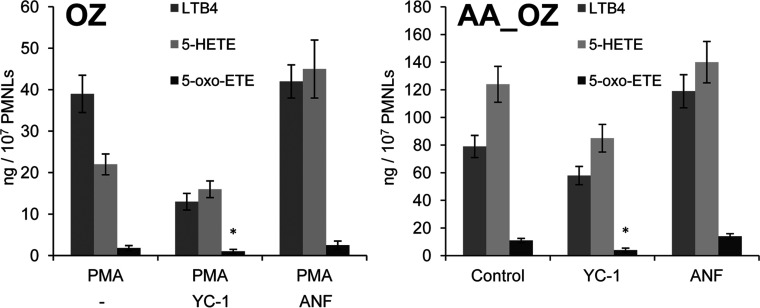
Influence of guanylate cyclase activators on 5-LOX product formation in OZ- and AA_OZ-stimulated PMNLs The cells (2×10^7^) were incubated for 30 min at 37°C without additives or with 2 nM PMA, 3 μM YC-1 or 30 nM ANF as indicated, followed by a 15-min incubation with 2 mg/ml OZ (**A**) or AA_OZ (16 μM AA) (**B**). The 5-LOX products were extracted from the medium and separated using HPLC. The results represent the means±S.D. of three independent experiments, *P<0.05 versus corresponding control.

The synthesis of 5-oxo-ETE requires the activation of 5-LOX and 5-HEDH (review in [26]). NO appears to activate and inhibit the formation of 5-oxo-ETE in neutrophils. NO-releasing compounds are potent activators of NO-sensitive sGC (soluble guanylyl cyclase) [27,28]. sGC and protein kinase G mediate NO suppression of 5-LOX metabolism in rat macrophages [29] and human neutrophils [19]. YC-1 is an NO-independent sGC activator [30]. The mGC (membrane-bound form of GC) is activated by a series of ligands, including ANF (atrial natriuretic peptide), but not by NO. We observed that adding YC-1 30 min before adding zymosan inhibited LTB_4_ and 5-oxo-ETE syntheses in PMNLs (Figure 4). ANF, an activator of mGC, increased the formation of LTB_4_ and 5-oxo-ETE in the presence of AA_OZ. Likewise, NO inhibited the activity of 5-LOX by 30–40% when added 5 min (Figure 3A) before OZ or 30 min (results not shown) before OZ or OS. Therefore we assume that the positive effect of adding NO last is related to its inability to inhibit 5-LOX when PMNLs commence phagocytosis.

The second enzyme involved in the synthesis of 5-oxo-ETE, i.e., 5-HEDH, requires NADP^+^ as a cofactor. NO induces a rapid decrease in intracellular NADPH levels [31]. We propose that NO activates 5-oxo-ETE formation in neutrophils through interactions of 5-LOX and 5-HEDH pathways that are regulated by numerous factors. Treatment of PMNLs with NO_OZ generated the maximal yield of 5-oxo-ETE from endogenously formed 5-HETE, compared with the yield in the absence of NO (Figures 1–3). PMNLs treated with NO efficiently converted available endogenously formed 5-HETE to 5-oxo-ETE.

**Figure 5:**
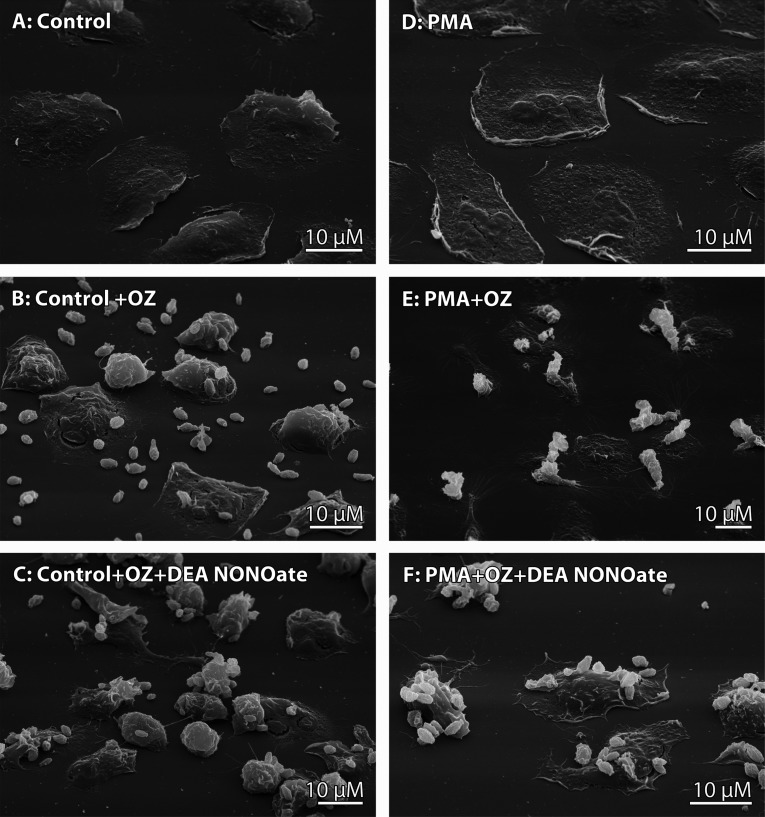
Effect of NO on the interaction of human neutrophils with OZ SEM images of human neutrophils that attached to fibronectin-coated substrata under the control conditions (**A**) or in the presence of 2 nM PMA (**D**) and were then exposed to opsonised zymosan (**B**,**E**) or DEA NONOate (final concentration, 500 μM NONOate) together with OZ (**C**,**F**). Images are representative of the results of three independent experiments.

DEA NONOate induced changes in 5-LOX and 5-HEDH activity that coincided with alterations in the interaction of human neutrophils with opsonised zymosan particles in the presence of the NO donor (Figure 5). Human neutrophils can phagocytose bacteria or OZ and bind them to their outer surface. We had previously demonstrated that extracellular binding occurs either by direct attachment of bacteria or yeast to the neutrophil surface or by binding of these cells at a distance through cytonemes (dynamic thread-like membrane tubulovesicular extensions of neutrophils [32]). Our data indicate that NO donors induced cytoneme formation in human neutrophils and shifted the interaction between neutrophils and bacteria or zymosan particles towards extracellular binding instead of phagocytosis [19,33,34]. SEM observations showed that treatment with DEA NONOate stimulated extracellular attachment of OZ particles to the cell surface (Figure 5) in the presence or absence of PMA.

## DISCUSSION

Neutrophils play a major role in the immune response to bacterial and fungal infections and eliminate pathogens through phagocytosis [3,35,36]. During phagocytosis, neutrophils produce ROS and release proteolytic enzymes that inflict local tissue damage. PMNLs undergo apoptosis and are removed from the site of inflammation. It was shown that dying neutrophils exhibit a dramatically increased ability to synthesize 5-oxo-ETE [37]. Here, we have shown that NO, a potent activator of apoptosis, uniquely induces 5-oxo-ETE synthesis in human neutrophils when they interact with their phagocytic target.

5-oxo-ETE synthesis increases in neutrophils and other phagocytic cells through activation of NADPH oxidase [16]. Oxidative stress increases 5-oxo-ETE formation in response to exogenously added 5-HETE [38]. Here, we observed a concomitant increase in LTB_4_ synthesis and release of 5-oxo-ETE when neutrophils interacted with OZ when exposed to PMA or LPS. The effect was highest when zymosan particles were added together with the NO-releasing compound DEA NONOate (Figures 2 and 3).

The synthesis of 5-oxo-ETE requires the activation of 5-LOX but also requires a stimulus that increases the activity of the highly specific enzyme 5S-hydroxyeicosanoid dehydrogenase that requires NADP^+^. NADP^+^ is normally present at low intracellular concentrations, which limits the basal synthesis of 5-oxo-ETE. G6PD (glucose-6-phosphate dehydrogenase) is the key enzyme that maintains intracellular levels of NADPH [39]. AA is an inhibitor of G6PD activity [39]. Moreover, AA activates NADPH oxidase in PMNLs, leading to superoxide formation and oxidative stress [22]. Our present findings show that AA_OZ induced a significant net release of 5-oxo-ETE in the absence of PMA (Figure 1C).

The NADPH level in cells is rapidly reduced by the addition of an NO donor [40]. NO is lipophilic and migrates through cells, which allows it to interact with numerous molecules [41]. One may propose that 5-oxo-ETE can be made from 5-HPETE by a free radical reaction (as reported for macrophages [42]). However, observed patterns of 5-oxo-ETE synthesis indicate contradiction with this hypothesis. We did treatments for stimulation of increased formation of 5-HPETE in cells. According to the data of Hatzelmann and Ullrich [24,25], Dnp-Cl and diamide, the agents lowering the GSH-peroxidase activity in cells, increased 5-HPETE level in neutrophils. We used Dnp-Cl and diamide in our experiments. We have found that Dnp-Cl and diamide rather inhibited than increased 5-oxo-ETE formation in PMNLs exposed to NO (results not shown).

When neutrophils are challenged with OZ or OS in the presence of an NO donor, they undergo a dramatic shift in 5-LOX metabolism that results in the formation of 5-oxo-ETE through a mechanism that remains to be defined in detail. Excessive and unregulated NO synthesis is implicated in many pathophysiological conditions, including cancer [43–45]. High neutrophil counts are associated with poor clinical outcomes for different types of cancer [46–49]. Systemic inflammation promotes the progression of malignancies through mechanisms mediated by neutrophils [50]. For example, treating neutrophils *in vitro* with 500 nM PMA significantly increases the adhesion of tumour cells to neutrophils [51].

NO is an important regulator of the interaction between macrophages and bacteria. For example, *Salmonella enterica* serovar *Enteritidis* effectively suppresses the production of NO by HD11 chicken macrophages [52], but an inhibitor of protein kinase A, H-89, dramatically reverses the suppressive effect of *Salmonella enteritidis* on the production of NO [53]. The bacteriostatic activity of H-89 augments the killing of *Salmonella enteritidis* by neutrophils [53]. We speculate that this effect may be mediated by 5-LOX metabolites and 5-oxo-ETE because the inhibition of protein kinase A by H-89 potentially activates 5-LOX [54].

In summary, we have shown here that exogenous and endogenous NO are intimately involved in the interaction between leucocytes and bacteria. NO plays a critical role in the synthesis of 5-oxo-ETE in neutrophils. In its turn, 5-oxo-ETE can enhance the survival of neutrophils by stimulating the release of GM-CSF (granulocyte/macrophage colony-stimulating factor) from monocytes [55]. During inflammation, neutrophil apoptosis can be markedly delayed by GM-CSF and G-CSF (granulocyte colony-stimulating factor) [56,57]. From other side, in PMNLs pretreated with GM-CSF or G-CSF, the 5-oxo-ETE becomes a potent activator of degranulation and superoxide anion production [58]. Tight regulation of the neutrophil lifespan is a critical process in resolving inflammation and the 5-oxo-ETE synthesis in neutrophil may be relevant to neutrophil survival.
